# Trends in stroke-related mortality in atrial fibrillation patients in the United States: Insights from the CDC WONDER database

**DOI:** 10.1016/j.ahjo.2024.100491

**Published:** 2024-12-06

**Authors:** Muhammad Abdullah Naveed, Sivaram Neppala, Himaja Dutt Chigurupati, Muhammad Omer Rehan, Ahila Ali, Hamza Naveed, Bazil Azeem, Rabia Iqbal, Manahil Mubeen, Mashood Ahmed, Ayman R. Fath, Timir Paul, Muhammad Bilal Munir

**Affiliations:** a Department of Cardiology, Dow Medical College, Dow University of Health Sciences, Karachi, Pakistan; b Division of Cardiology, The University of Texas Health Sciences Center, San Antonio, TX, USA; c Department of Internal Medicine, New York Medical College at Saint Michael's Medical Center, Newark, NJ 07102, USA; d Department of Internal Medicine, Queen Elizabeth the Queen Mother Hospital, EKHUFT, Margate, Kent, United Kingdom; e Department of Cardiology, Shaheed Mohtarma Benazir Bhutto Medical College Lyari, Karachi, Pakistan; f Department of Medicine, Rawalpindi Medical University, Rawalpindi, Pakistan; g Department of Cardiovascular Sciences, Ascension St. Thomas Hospital, University of Tennessee Health Sciences Center, Nashville, TN, USA; h Department of Cardiac Electrophysiology, University of California Davis Health, Sacramento, CA, USA

**Keywords:** Atrial fibrillation, Stroke, Age-adjusted mortality rates, Race, Ethnicity, Sex, Geographic regions

## Abstract

**Background:**

Stroke associated with atrial fibrillation (AF) is a significant cause of mortality. This study analyzed demographic trends and disparities in mortality rates due to stroke in AF patients aged ≥25 years.

**Methods:**

A retrospective analysis was conducted to acquire death data using the Centers for Disease Control and Prevention database from 1999 to 2020. Age-adjusted mortality rates (AAMRs) were calculated per 100,000 persons, and trends were assessed using Average Annual Percentage Change (AAPC) and annual percent change (APC). Data were stratified by year, sex, race/ethnicity, and geographical regions.

**Results:**

Between 1999 and 2020, AF-associated stroke contributed to 331,106 deaths among adults in this study population. Deaths occurred predominantly in medical facilities (43.2 %). The overall AAMR for AF-associated stroke decreased from 7.4 in 1999 to 6.4 in 2020, with an APC of −1.02 (*p*-value = 0.004). Additionally, AAMR showed a significant decline from 2015 to 2018 with an APC of −7.22 (p-value <0.000001), followed by a striking rise from 2018 to 2020 (APC: 4.98) (p-value = 0.0008). Women had slightly higher AAMR than men (men: 6.6; women: 7.1) (*p* value = 0.02). AAMRs varied among racial/ethnic groups, with Whites having the highest AAMR (7.4), followed by Blacks (5.4), American Indian or Alaska Natives (4.6), Asian or Pacific Islanders (4.5), and Hispanics (4.1). AAMRs decreased for all races except Blacks. Geographically, AAMRs ranged from 4.3 in Nevada to 11.9 in Vermont, with the Western region showing the highest mortality (AAMR: 7.9). Nonmetropolitan areas had slightly higher AAMRs than metropolitan areas, with both experiencing a decrease over the study period.

**Conclusion:**

This analysis depicts significant demographic and geographic disparities in mortality rates attributed to stroke associated with AF. Targeted interventions and equitable healthcare access are crucial to mitigate these disparities and improve outcomes for this population.

## Highlights

• The overall Age-Adjusted Mortality Rate (AAMR) associated with strokes from atrial fibrillation (AF), declined from 7.4 in 1999 to 6.4 in 2020.
• A higher AAMR was observed in adult women compared to men, with women demonstrating a decreasing trend from 1999 to 2020.
• From 1999 to 2020, both Asian and white populations experienced a decrease in mortality rates, while no mortality rates of other races showed no variation.
• Western states and Nonmetropolitan areas demonstrated notably higher mortality rates than other states and areas.

## 1 Introduction

Stroke stands as a significant global threat to mortality and quality of life, particularly when associated with atrial fibrillation (AF), a significant public health challenge [[1], [2], [3], [4]]. AF, as the most prevalent sustained cardiac arrhythmia, affects millions worldwide and escalates the risk of stroke by fivefold [[5], [6], [7]]. Emerging evidence indicates that the risk of ischemic stroke escalates in elderly patients with AF, rising from 4.6 % at ages 50–59 years to 20.2 % at ages 80–89 years, which calls for urgent attention [8,9]. Numerous studies have indicated that ischemic stroke related to AF is linked to a notable risk of mortality, longer hospitalizations, and poorer functional outcomes [[10], [11], [12], [13]]. Over the past two decades, we have witnessed significant strides in preventing AF-related strokes by anticoagulation use and managing risk factors [14,15]. In 2019, the American College of Cardiology (ACC)/The American Heart Association (AHA) updated guidelines recommending oral anticoagulation for individuals with AF and CHA2DS2-VASc scores ≥2 in men and ≥ 3 in women [16].

Variations in geographic location within the United States significantly influence the outcomes of stroke and AF. Regions such as the southeastern U.S., also known as the “Stroke Belt,” display higher incidence of stroke and related mortality [17]. This inequality is commonly attributed to variations in healthcare accessibility, socioeconomic factors, and the prevalence of comorbid conditions like hypertension and diabetes. Moreover, gender and racial disparities significantly influence the risk and outcomes of stroke in AF patients. While men generally face a higher risk of developing AF, recent studies have revealed a 1.3-fold increased risk of stroke in women with AF, even among anticoagulated patients, with women experiencing a higher annual risk rate of 2.4 % compared to men [[18], [19], [20]].

Furthermore, racial disparities are also apparent. Previous studies have indicated a decline in stroke incidence in the white population. In contrast, ischemic stroke incidence in the black population remains unchanged even after stratification by race and stroke subtype [21,22]. These compelling findings underline the critical need to prioritize a comprehensive understanding of the relationship between stroke and AF, particularly considering the disparities in mortality rates associated with these conditions.

Strokes associated with AF should be recognized as a distinct clinical entity that warrants dedicated research, separate from the broader category of ischemic and hemorrhagic strokes. The pathophysiology, risk factors, and recurrence patterns of AF-related strokes are significantly different from those of other stroke subtypes, indicating specific therapeutic requirements and implications. The embolic strokes associated with AF often necessitate customized anticoagulation strategies that are not universally applicable to all ischemic stroke patients. By investigating AF-related strokes as a separate category, we may enhance the precision of therapeutic interventions and ultimately improve patient outcomes. This study utilizes data from the CDC WONDER database from 1999 to 2020 to address these issues and better understand the mortality trends associated with stroke in AF patients. We aim to identify and characterize demographic trends and disparities in mortality rates among AF patients aged 25 and older.

## 2 Methods

### 2.1 Study design

The study sourced data from the CDC WONDER (Centers for Disease Control and Prevention) database, a highly reliable and comprehensive repository of death certificates from all 50 states and the District of Columbia from 1999 to 2020.

This study utilized de-identified, publicly available datasets issued by the government and voluntarily adhered to the Strengthening the Reporting of Observational Studies in Epidemiology (STROBE) guidelines for reporting. Due to the nature of the data, institutional review board approval (IRB) was optional.

### 2.2 Study cohort

The study included adults aged 25 years or older diagnosed with atrial fibrillation between 1999 and 2020. We examined death records from the Multiple Causes of Death Public Use registry to identify stroke-related mortality in these patients. Stroke was defined as any type of stroke, including ischemic, hemorrhagic, or both. Stroke-related mortality was either the primary cause of death or a contributing factor. The cohort was identified using the International Classification of Diseases (ICD) codes, which identified our cohort as follows: I48 (Atrial fibrillation) and I60–69 (stroke).

### 2.3 Data extraction

Demographic data, including age, gender, and race/ethnicity, were extracted, along with information on population size, urban-rural stratification, regional delineation, state-specific classification, and year and location of death. The location of death was categorized into medical facilities (outpatient, emergency room, inpatient, death on arrival, or status unknown), home, hospice, and nursing home/long-term care facility. Race/ethnicity was classified into Hispanic and non-Hispanic White, African American, Asian, or Pacific Islanders.

Population assessment was conducted using the National Center for Health Statistics Urban-Rural Classification Scheme to define urban (large central metropolitan, large fringe metropolitan, medium metropolitan, and small metropolitan) and nonmetropolitan (micropolitan and noncore) counties according to the 2013 US census classification for reporting the place of death. Additionally, based on the 2010 US Census Bureau definitions, regions were categorized into Northeast, Midwest, South, and West.

### 2.4 Statistical analysis

The crude and Age-Adjusted Mortality Rate (AAMR) per 100,000 individuals was calculated to investigate nationwide mortality trends. This involved the determination of the total number of fatalities attributed to stroke in the population with AF for each year. As per standard practice, the AAMR was calculated by standardizing the Stroke-related deaths using the 2000 US population and 95 % confidence intervals CI). The JoinPoint Regression Program (Joinpoint V 4.9.0.0, National Cancer Institute, Bethesda, MD, USA) determined the annual percent change (APC) and a 95 % CI in AAMR. AAMRs were employed to equitably compare mortality rates across different populations or historical periods. By analyzing AAMRs, the study was able to discern mortality patterns and identify significant fluctuations over time by utilizing log-linear regression models.

## 3 Results

Between 1999 and 2020, Stroke in AF patients accounted for a total of 331,106 deaths among adults aged 25 years and above in the United States (Supplementary Table 1). These fatalities were distributed across various settings, with the leading most occurring in medical facilities (43.2 %), 31.8 % in nursing homes/long-term care facilities, 15.6 % at the decedents' homes, 5.6 % in hospice facilities, and 3.7 % at other locations (Supplementary Table 2). The central illustration summarizing the study's characteristics and findings is presented in Fig. 5.

### 3.1 Annual Trends for Stroke in AF-Related Age-Adjusted Mortality Rate (AAMR)

The age-adjusted mortality rate (AAMR) for Stroke in AF-related deaths among adults has shown a significant decrease from 7.4 in 1999 to 6.4 in 2020, with an Average Annual Percentage Change (AAPC) of −1.02 (95 % Confidence Interval [CI]: −1.55 to −0.53) (*p*-value = 0.004). Notably, there was a significant decline in AAMR from 2015 to 2018 (APC: -7.22; 95 % CI: −8.86 to −4.99) (*p*-value <0.000001), but no significant changes were noted from 1999 to 2015 (APC: -0.27; 95 % CI: −0.63 to 0.10) (p-value = 0.11). Lastly, there was a striking rise in AAMR from 2018 to 2020 (APC: 4.98; 95 % CI: 1.66 to 7.99) (p-value = 0.0008). (Supplementary Table 3).

### 3.2 Stroke in AF-related AAMR stratified by sex

**Fig. 1:**
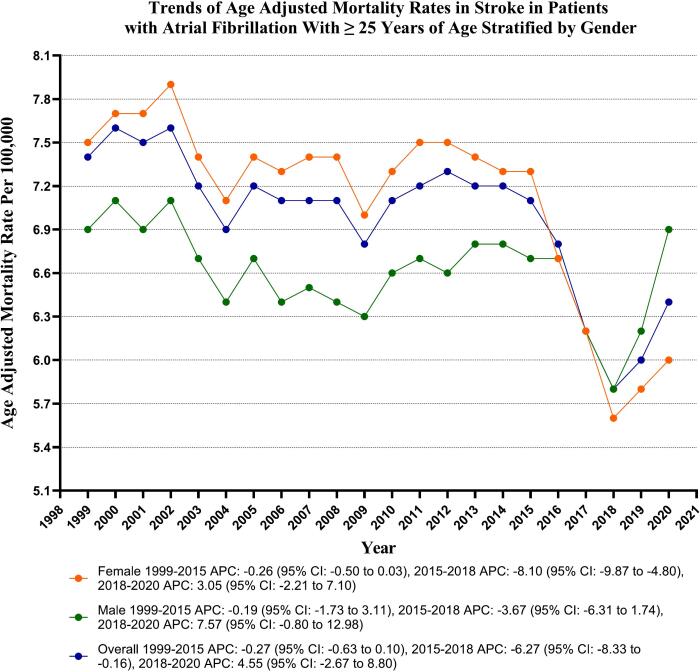
Overall and Sex-Stratified Stroke related age-adjusted mortality rates per 100,000 in Adults with Atrial Fibrillation in the United States, 1999 to 2020.

Throughout the study, adult women exhibited slightly higher AAMRs than adult men (overall AAMR for men: 6.6, 95 % CI: 6.6–6.6; for women: 7.1, 95 % CI: 7.0–7.1). The AAMR for adult men showed variable trends, and it has demonstrated decreased trends from 2015 to 2018 (APC: -5.40; 95 % CI: −7.26 to −3.17, *p*-value <0.000001); similar trends were noted in the females (APC: -8.10; 95 % CI: −9.86 to −4.80, *p*-value = 0.01). Whereas the male gender has shown increasing trends of mortality from 2018 to 2020 (APC: 8.35; 95 % CI: 4.59 to 11.72, p-value <0.000001), but the female gender showed no significant difference in the same period (APC: 3.04; 95 % CI: −2.21 to 7.10, p-value = 0.19). (Supplementary Table 4 and Fig. 1).

### 3.3 Stroke in AF-related AAMR in AF patients stratified by race/ethnicity

Significant variability in mortality rates was found among different racial/ethnic groups, with the highest mortality occurring in White patients (289,277 deaths; 87.4 %), followed by Black patients (20,835 deaths; 6.3 %), Hispanic patients (12,333 deaths; 3.7 %), Asian or Pacific Islander patients (7170 deaths; 2.2 %), and the lowest number in American Indian or Alaska Native patients (930 deaths; 0.3 %). AAMRs were highest among Whites, followed by Black or African Americans, American Indian or Alaska Natives, Asian or Pacific Islanders, and Hispanic or Latinos (overall AAMR: White: 7.4, 95 % CI: 7.4–7.4; Black or African American: 5.4, 95 % CI: 5.3–5.4; American Indian or Alaska Native: 4.6, 95 % CI: 4.3–4.9; Asian or Pacific Islander: 4.5, 95 % CI: 4.4–4.6; Hispanic or Latino: 4.1, 95 % CI: 4.1–4.2).

**Fig. 2:**
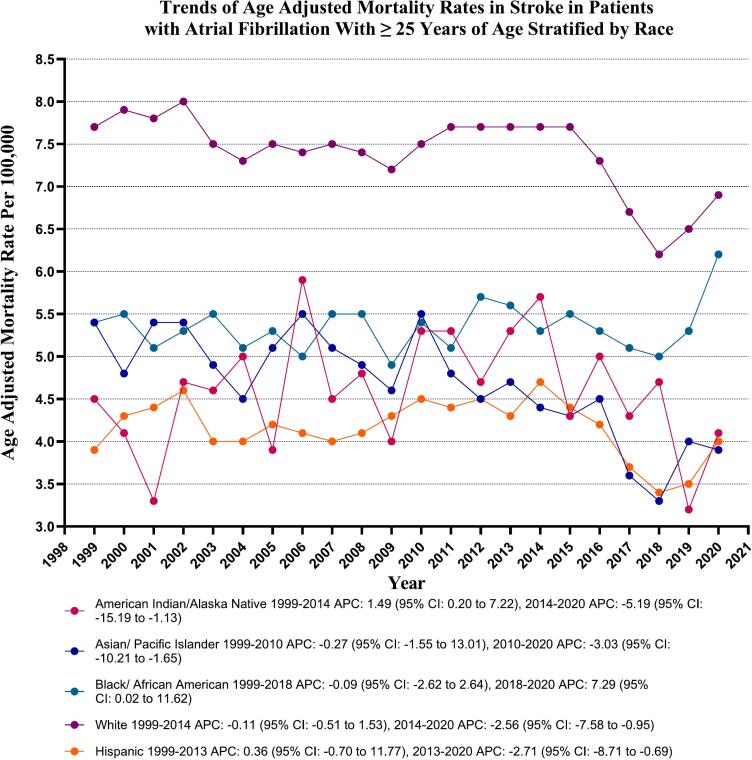
Stroke-related age-adjusted mortality rates per 100,000 Stratified by Race in Adults with Atrial fibrillations in the United States, 1999 to 2020.

The AAMR of the Asian and White populations exhibited a decreasing trend from 1999 to 2020. Specifically, the AAPC for Asians was −1.60 (95 % CI: −2.50 to −0.24, *p*-value = 0.02), and for Whites, it was −0.82 (95 % CI: −1.35 to −0.33, *p*-value = 0.01). However, no significant changes were observed in the AAMR of Hispanic, American Indian, and Black populations during the same period. The AAPC for Hispanics was −0.67 (95 % CI: −1.33 to 0.52, p-value = 0.39); for Americans, it was −0.46 (95 % CI: −1.55 to 1.14, p-value = 0.76), and for Blacks, it was 0.59 (95 % CI: −0.11 to 0.98, p-value = 0.12). (Supplementary Table 5 and Fig. 2).

### 3.4 Stroke in AF-related AAMR stratified by geographical regions

Variations in AAMRs were observed among different states, with AAMRs ranging from as low as 4.3 (95 % CI: 4.1–4.6) in Nevada up to 11.9 (95 % CI: 11.3–12.6) in Vermont. States falling within the top 90th percentile included Alaska, Oregon, Rhode Island, Vermont, Washington, and West Virginia, which had approximately 1.5 times higher AAMRs compared to states in the lower 10th percentile, which included Arizona, Florida, Georgia, Kansas, Louisiana, Nevada, New Mexico, and New York. (Supplementary Table 6).

**Fig. 3:**
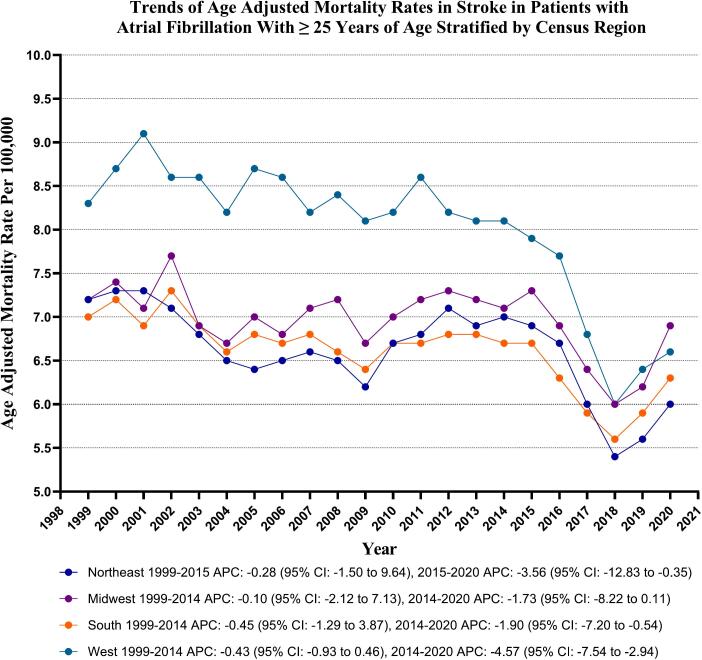
Stroke-related age-adjusted mortality rates per 100,000 Stratified by Regions in Adults (≥25 Years) with Atrial Fibrillation in the United States, 1999 to 2020.

On average, over the study period, the highest mortality was observed in the Western (AAMR: 7.9; 95 % CI: 7.8 to 8.0), followed by Midwestern (AAMR: 7.0; 95 % CI: 6.9 to 7.0), Northeastern (AAMR: 6.6; 95 % CI: 6.5 to 6.6), and Southern regions (AAMR: 6.6; 95 % CI: 6.5 to 6.6). (Supplementary Table 7 and Fig. 3).

**Fig. 4:**
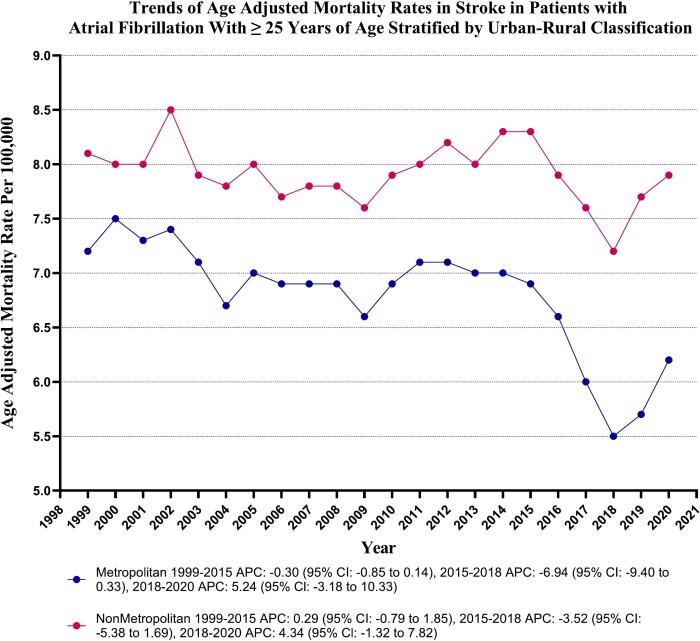
Stroke-related age-adjusted mortality rates per 100,000 Stratified by Urbanization in Adults (≥25 Years) with Atrial Fibrillation in the United States, 1999 to 2020.

In the duration of the study, nonmetropolitan areas consistently displayed slightly higher Age-Adjusted Mortality Rates (AAMRs) compared to metropolitan areas, with overall AAMRs of 7.9 (95 % CI: 7.8 to 8.0) and 6.8 (95 % CI: 6.7 to 6.8) respectively. The AAMR of metropolitan areas experienced a decline from 1999 to 2020 [Metropolitan: Annual Percent Change (APC): -0.89, (CI: −1.17 to −0.69) (*p*-value <0.000001)]. Conversely, the nonmetropolitan regions did not exhibit a statistically significant trend during the same period [Nonmetropolitan: APC: -0.09, (CI: −0.31 to 0.11) (p-value = 0.35)]. (Supplementary Table 8 and Fig. 4).

**Fig. 5:**
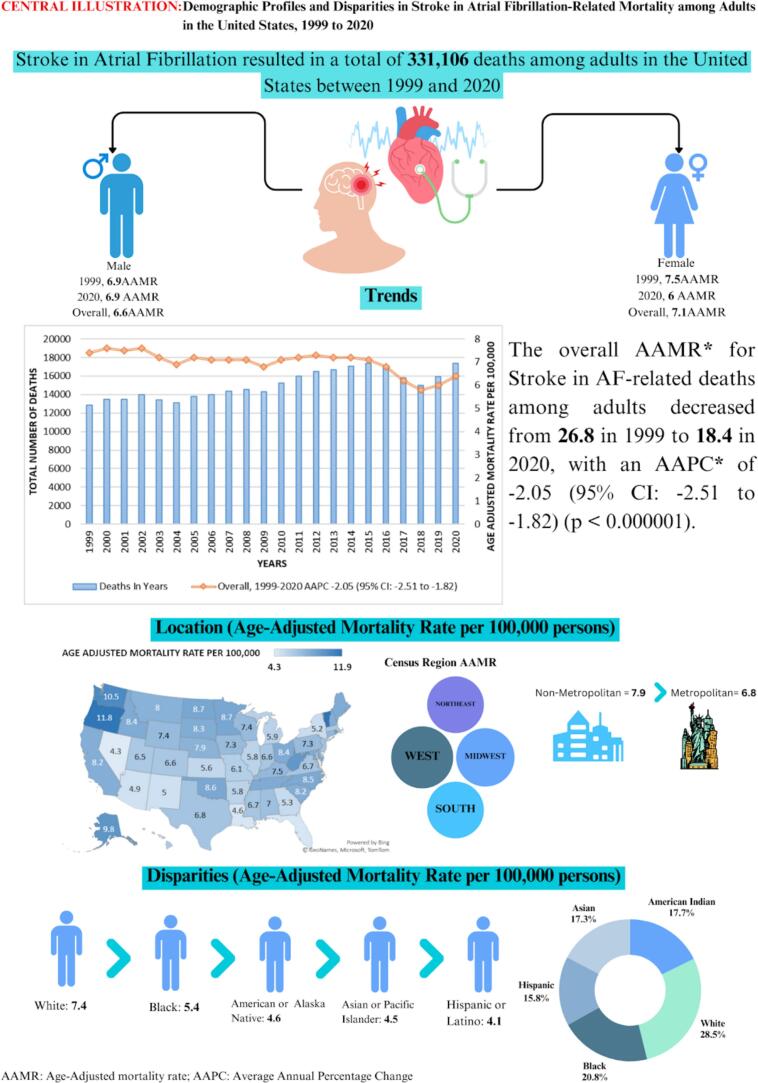
Central Illustration: Trends in Demographics and Disparities in Stroke-Related Mortality in Atrial Fibrillation Patients in the United States: 1999 to 2020.

## 4 Discussion

In this comprehensive 20-year analysis of mortality data from the Centers for Disease Control and Prevention in the United States, we have uncovered several crucial findings regarding the impact of AF on stroke mortality:

1. The age-adjusted mortality rate for stroke in AF-related deaths among adults decreased from 1999 to 2020, with an overall annual reduction of 1.02 %. The decline was significant between 2015 and 2018 but stable from 1999 to 2015. From 2018 to 2020, there was a notable increase in mortality rates. A higher age-adjusted mortality rate was observed in adult women compared to men, with women demonstrating a decreasing trend from 1999 to 2020. Conversely, no significant difference in mortality rate was noted in men during the same period.
2. From 1999 to 2020, both Asian and white populations experienced a decrease in mortality rates, while no significant variance was observed in the mortality rates of Hispanic, American Indian, and Black populations during the same period.
3. Our analysis indicates elevated mortality rates in the western states, followed by the midwestern states. Nonmetropolitan areas demonstrated notably higher mortality rates than metropolitan areas. We observed declining mortality trends in metropolitan areas, whereas nonmetropolitan regions did not exhibit statistically significant trends.

Our findings demonstrate a reduction in the age-adjusted mortality rate (AAMR) attributed to stroke in patients with AF from 1999 to 2018 and have noted a significant rise in mortality trends from 2018 to 2020. This decline is consistent with prior investigations, highlighting considerable progress and developments in medical interventions aimed at preventing strokes in AF patients, mainly through anticoagulants [[14], [15], [16]]. These advancements have had a notable positive impact on patient outcomes and offer a promising prospect for the future. However, from 2018 to 2020, we observed a reversal in this mortality trend, which was also statistically significant; this recent uptick can be attributed to various factors, including the impact of the COVID-19 pandemic and the growing prevalence of comorbidities such as obesity, diabetes, and chronic kidney diseases in adults, all directly linked to AF and stroke mortality [[23], [24], [25], [26]].

Numerous studies have been conducted to examine sex differences in stroke, revealing inconsistent findings concerning the mortality rates associated with stroke in women. Some research has indicated a higher incidence of stroke and venous thromboembolism in women, coupled with an elevated mortality rate compared to men. For instance, Wang et al. analyzed patients from the Framingham Heart Study, revealing a 1.6-fold higher risk of mortality in females compared to males [27]. Similarly, findings from Dagres et al. in the Euro Heart Study, who investigated gender-related differences in adult patients with AF in Europe, demonstrated that women had an increased risk of stroke-related mortality with 1.8–1.9-fold and had higher comorbidities compared to men [28]. Another study by Friberg et al. in the Swedish study found that comorbidities, including prior myocardial infarction, vascular disease, and renal failure, predict ischemic stroke and composite thromboembolism endpoints in AF patients [29]. Our study supported these results, indicating a higher overall mortality rate among women compared to men (age-adjusted mortality rate: 7.1 % vs. 6.6 %). Women experience unique changes throughout their lifespans, such as pregnancy, hormonal changes, and exogenous hormone infusion, which may impact the vascular system. Also, women have increased odds of receiving nonoptimal anticoagulation, as was demonstrated by Eckman and his colleagues [30]. Importantly, our research identified a substantial reduction in mortality rates for both men and women from 2015 to 2018, likely driven by the growing adoption of direct oral anticoagulants (DOACs). These medications have been pivotal in decreasing stroke risk among patients with AF, including women. Nevertheless, despite these advancements, women with AF face a notably higher risk of stroke compared to men, a disparity highlighted in recent studies [31]. This underscores the urgent need for tailored management strategies to tackle these gender-specific challenges. It is crucial to consider these factors when assessing risk and developing prevention plans for women.

Moreover, further research is essential to uncover the root causes of the elevated stroke risk in women, particularly post-menopause, and to investigate targeted interventions that could enhance vascular function and prevent strokes. These factors must be considered when evaluating risk and formulating prevention plans for women. There is a pressing need for additional research to pinpoint the causes of the heightened risk for women, particularly post-menopause, and to investigate the potential targeting of factors that impact vascular function for stroke prevention.

Our research has uncovered significant disparities in stroke mortality across various racial and ethnic groups, highlighting the pressing need to confront persistent health inequities. The Caucasian/White population exhibited the highest AAMR at 7.4 %, followed by individuals of Black ethnicity at 5.4 %. The AAMR displayed a declining trend in the Asian and Caucasian populations from 1999 to 2020. However, no significant variances in AAMR were observed among Hispanic, Black, and American Indian populations. Prior research suggests that individuals of Black or African American descent experience a rising trend in stroke-related mortality, possibly due to a higher prevalence of risk factors such as diabetes, hypertension, and renal diseases, which increase the risk of stroke [32,33]. These differences in stroke incidence are the primary drivers of the disparities in stroke mortality rates.

Significant variations in mortality rates were observed geographically, with the Western region recording the highest AAMR (7.9) and Nevada the lowest (4.3). Previous studies have highlighted that southeastern states exhibit 2–4 times higher risks than others and have been identified as a ‘stroke belt’ for several decades [34]. Furthermore, nonmetropolitan areas demonstrated notably higher mortality rates than metropolitan areas. We observed declining mortality trends in metropolitan areas, whereas nonmetropolitan regions did not exhibit statistically significant trends. These geographical and regional disparities underscore the importance of localized factors and access to healthcare in influencing stroke mortality rates. This emphasizes the need for interventions and resource allocation tailored to specific regions to ensure the most effective and targeted approach to reducing disparities.

While progress has been made in reducing the mortality trends for AF-associated stroke, persistent disparities and recent fluctuations underscore the need for continued research and targeted public health strategies. It is crucial to address gender, racial, and geographic disparities to improve outcomes further and ensure equitable healthcare for all populations.

## 5 Limitation

The study has limitations, mainly due to its retrospective design. Relying on death certificates in the CDC WONDER database introduces the potential for inaccurate diagnosis, leading to misclassification bias. Furthermore, the absence of laboratory values, medication lists, and clinical data about general health conditions, comorbidities, and treatment limits a comprehensive understanding of mortality patterns. Nevertheless, compared to current literature, this study includes adults aged 25 and older from all racial backgrounds, providing a thorough analysis of stroke-related mortality trends across a diverse population. Covering the period from 1999 to 2020, our study offers a long-term perspective that enhances our understanding of the evolution of stroke mortality over more than two decades.

## 6 Conclusion

The analysis reveals notable demographic and geographic disparities in mortality rates linked to stroke and AF. While mortality rates have generally declined, recent data indicate a heightened necessity for extended monitoring to ascertain whether this trend will continue or decrease. Specific interventions and equitable healthcare access must be deployed to mitigate these disparities and enhance outcomes for this demographic.

## CRediT authorship contribution statement

**Muhammad Abdullah Naveed:** Writing – original draft, Methodology, Formal analysis. **Sivaram Neppala:** Writing – review & editing, Writing – original draft, Supervision, Investigation. **Himaja Dutt Chigurupati:** Writing – original draft, Methodology. **Muhammad Omer Rehan:** Visualization, Validation. **Ahila Ali:** Writing – original draft, Formal analysis. **Hamza Naveed:** Resources, Methodology. **Bazil Azeem:** Writing – original draft, Formal analysis, Data curation. **Rabia Iqbal:** Writing – original draft, Data curation. **Manahil Mubeen:** Formal analysis, Writing – original draft. **Mashood Ahmed:** Visualization, Validation, Data curation. **Ayman R. Fath:** Writing – review & editing, Supervision. **Timir Paul:** Writing – review & editing, Supervision, Project administration. **Muhammad Bilal Munir:** Writing – review & editing, Supervision, Resources, Project administration.

## Ethics approval and consent to participate

Not Applicable.

## Funding

The authors received no extramural funding for the study.

## Declaration of competing interest

The authors declare that they have no known competing financial interests or personal relationships that could have appeared to influence the work reported in this article.

## Appendix A Supplementary data

Supplementary tables Image 1
