# Supplementary material for: Trends in stroke-related mortality in atrial fibrillation patients in the United States: Insights from the CDC WONDER database
Source: Am Heart J Plus. 2024 Dec 6;49:100491. doi: 10.1016/j.ahjo.2024.100491 (PMC11696626; doi:10.1016/j.ahjo.2024.100491)
Supplement: Supplementary file 1 — Supplementary tables [file mmc1.docx]

**Supplement tables:**

**Supplemental Table 1: Stroke-related mortalities in patients with Atrial Fibrillation, Stratified by Sex and Race, in Older Adults in the United States, 1999 to 2020**

| **Year** | **Overall** | **Women** | **Men** | **NH White** | **NH Black or African American** | **NH Asian or Pacific Islander** | **NH American Indian or Alaska Native** | **Hispanic or Latino** |
| --- | --- | --- | --- | --- | --- | --- | --- | --- |
| 1999 | 12889 | 8576 | 4313 | 11638 | 738 | 184 | 26 | 271 |
| 2000 | 13468 | 8946 | 4522 | 12176 | 757 | 171 | 25 | 315 |
| 2001 | 13509 | 9029 | 4480 | 12185 | 715 | 205 | 22 | 351 |
| 2002 | 13997 | 9338 | 4659 | 12598 | 747 | 224 | 28 | 374 |
| 2003 | 13425 | 8941 | 4484 | 12008 | 800 | 214 | 30 | 346 |
| 2004 | 13104 | 8681 | 4423 | 11736 | 745 | 205 | 35 | 368 |
| 2005 | 13827 | 9126 | 4701 | 12330 | 791 | 258 | 29 | 405 |
| 2006 | 13958 | 9272 | 4686 | 12417 | 758 | 295 | 44 | 428 |
| 2007 | 14357 | 9501 | 4856 | 12730 | 857 | 289 | 34 | 439 |
| 2008 | 14572 | 9646 | 4926 | 12871 | 877 | 293 | 38 | 476 |
| 2009 | 14282 | 9306 | 4976 | 12590 | 822 | 291 | 32 | 519 |
| 2010 | 15223 | 9891 | 5332 | 13328 | 905 | 357 | 43 | 569 |
| 2011 | 15983 | 10362 | 5621 | 14063 | 898 | 348 | 46 | 609 |
| 2012 | 16497 | 10723 | 5774 | 14366 | 1044 | 351 | 44 | 663 |
| 2013 | 16697 | 10647 | 6050 | 14505 | 1043 | 397 | 53 | 667 |
| 2014 | 17044 | 10701 | 6343 | 14721 | 1041 | 407 | 57 | 781 |
| 2015 | 17355 | 10927 | 6428 | 14895 | 1128 | 424 | 51 | 801 |
| 2016 | 16874 | 10266 | 6608 | 14394 | 1142 | 457 | 59 | 791 |
| 2017 | 15774 | 9556 | 6218 | 13420 | 1125 | 404 | 56 | 740 |
| 2018 | 14978 | 8883 | 6095 | 12636 | 1149 | 384 | 65 | 717 |
| 2019 | 15916 | 9301 | 6615 | 13339 | 1242 | 495 | 49 | 773 |
| 2020 | 17377 | 9732 | 7645 | 14331 | 1511 | 517 | 64 | 930 |
| **Total** | **331,106** | **211,351** | **119,755** | **289,277** | **20,835** | **7,170** | **930** | **12,333** |

**Supplemental Table 2: Stroke-related Mortality, Stratified by Place of Death in Older Adults with Atrial Fibrillation in the United States, 1999 to 2020**

| **Deaths** | | | | |
| --- | --- | --- | --- | --- |
| **Year** | **Medical Facility** | **Nursing Home/Long-term Care Facility** | **Hospices** | **Home** |
| 1999 | 6,569 | 4853 | - | 1216 |
| 2000 | 6,770 | 4942 | - | 1448 |
| 2001 | 6,657 | 5024 | - | 1454 |
| 2002 | 6,775 | 5110 | - | 1650 |
| 2003 | 6,382 | 4831 | 27 | 1637 |
| 2004 | 6,200 | 4625 | 63 | 1635 |
| 2005 | 6,387 | 4909 | 213 | 1761 |
| 2006 | 6,423 | 4836 | 385 | 1781 |
| 2007 | 6,537 | 4761 | 505 | 2005 |
| 2008 | 6,499 | 4682 | 659 | 1971 |
| 2009 | 6,250 | 4491 | 653 | 2063 |
| 2010 | 6,554 | 4781 | 888 | 2348 |
| 2011 | 6,696 | 4961 | 1071 | 2535 |
| 2012 | 6,759 | 4899 | 1349 | 2704 |
| 2013 | 6,767 | 4971 | 1306 | 2826 |
| 2014 | 6,726 | 5143 | 1605 | 2950 |
| 2015 | 6,846 | 5091 | 1833 | 3026 |
| 2016 | 6,679 | 4707 | 1723 | 3141 |
| 2017 | 5,886 | 4711 | 1634 | 2991 |
| 2018 | 5,599 | 4380 | 1458 | 3029 |
| 2019 | 6,276 | 4317 | 1583 | 3222 |
| 2020 | 6,866 | 4127 | 1528 | 4137 |
| **Total** | **143,103** | **105,152** | **18,483** | **51,530** |

**Supplemental Table 3: Annual percent change (APC) of Stroke related Age-Adjusted Mortality Rates per 100,000 in Older Adults with Atrial Fibrillation in the United States, 1999 to 2020**

| **Year Interval** | **APC (95% CI)** | **P-value** |
| --- | --- | --- |
| **Overall** | | |
| 1999 to 2007 | -0.95 (-2.45 to -0.46) | < 0.000001 |
| 2007 to 2015 | 0.39 (-0.07 to 1.89) | 0.103179 |
| 2015 to 2018 | -7.22 (-8.86 to -4.99) | < 0.000001 |
| 2018 to 2020 | 4.98 (1.66 to 7.99) | 0.0008 |
| **Men** | | |
| 1999 to 2008 | -1.15 (-2.30 to -0.59) | < 0.000001 |
| 2008 to 2015 | 1.12 (0.43 to 3.38) | 0.0016 |
| 2015 to 2018 | -5.40 (-7.26 to -3.17) | < 0.000001 |
| 2018 to 2020 | 8.35 (4.59 to 11.72) | < 0.000001 |
| **Women** | | |
| 1999 to 2015 | -0.26 (-0.50 to 0.03) | 0.073985 |
| 2015 to 2018 | -8.10 (-9.86 to -4.80) | 0.019196 |
| 2018 to 2020 | 3.04 (-2.21 to 7.10) | 0.185163 |
| **NH White** | | |
| 1999 to 2008 | -0.75 (-1.64 to -0.35) | < 0.000001 |
| 2008 to 2015 | 0.89 (0.31 to 2.34) | 0.0016 |
| 2015 to 2018 | -7.40 (-8.82 to -5.39) | < 0.000001 |
| 2018 to 2020 | 5.32 (2.40 to 8.16) | 0.0004 |
| **NH Black or African American** | | |
| 1999 to 2018 | -0.09 (-2.62 to 2.64) | 0.441512 |
| 2018 to 2020 | 7.29 (0.02 to 11.62) | 0.04839 |
| **NH American Indian or Alaska Native** | | |
| 1999 to 2014 | 1.49 (0.20 to 7.22) | 0.026395 |
| 2014 to 2020 | -5.19 (-15.19 to -1.13) | 0.003199 |
| **Hispanic or Latino** | | |
| 1999 to 2015 | 0.53 (0.01 to 1.35) | 0.046791 |
| 2015 to 2018 | -9.23 (-11.85 to -4.26) | 0.016397 |
| 2018 to 2020 | 8.46 (1.75 to 13.95) | 0.027195 |
| **NH Asian or Pacific Islander** | | |
| 1999 to 2010 | -0.27 (-1.55 to 13.01) | 0.953809 |
| 2010 to 2020 | -3.03 (-10.21 to -1.65) | 0.003999 |
| **Nonmetropolitan areas** | | |
| 1999 to 2009 | -0.47 (-1.29 to -0.01) | 0.039192 |
| 2009 to 2015 | 1.28 (0.70 to 2.81) | < 0.000001 |
| 2015 to 2018 | -4.61 (-5.85 to -3.03) | < 0.000001 |
| 2018 to 2020 | 4.79 (2.37 to 7.04) | 0.0012 |
| **Metropolitan area** | | |
| 1999 to 2007 | -1.03 (-2.67 to 0.50) | < 0.000001 |
| 2007 to 2015 | 0.42 (-0.08 to 2.06) | 0.108378 |
| 2015 to 2018 | -7.96 (-9.80 to -5.52) | < 0.000001 |
| 2018 to 2020 | 5.72 (2.05 to 9.09) | < 0.000001 |
| **Northeast region** | | |
| 1999 to 2006 | -2.12 (-4.14 to -1.04) | 0.008798 |
| 2006 to 2015 | 1.19 (0.51 to 3.12) | 0.017197 |
| 2015 to 2018 | -8.36 (-10.80 to -4.90) | 0.020396 |
| 2018 to 2020 | 4.41 (-1.34 to 8.92) | 0.094381 |
| **South region** | | |
| 1999 to 2009 | -0.80 (-2.30 to -0.46) | 0.009598 |
| 2009 to 2015 | 0.48 (-0.20 to 2.55) | 0.167566 |
| 2015 to 2018 | -6.42 (-8.12 to -4.26) | < 0.000001 |
| 2018 to 2020 | 6.46 (2.91 to 9.82) | 0.0012 |
| **Midwest region** | | |
| 1999 to 2020 | -0.42 (-0.85 to 0.01) | 0.056789 |
| **West region** | | |
| 1999 to 2015 | -0.43 (-0.77 to 0.12) | 0.091182 |
| 2015 to 2018 | -8.70 (-11.06 to -0.66) | 0.026395 |
| 2018 to 2020 | 3.48 (-4.33 to 8.21) | 0.155969 |
| APC = annual percent change; NH = non-Hispanic; * Indicates that the annual percentage change (APC) is significantly different from zero at α = 0.05. AAMR = age-adjusted mortality rate. | | |

**Supplemental Table 4: Overall and Sex‐Stratified Stroke related Age-Adjusted Mortality Rates per 100,000 in Older Adults with Atrial Fibrillation in the United States, 1999 to 2020**

| **Age-Adjusted Rate (95% CI)** | | | |
| --- | --- | --- | --- |
| **Year** | **Men** | **Women** | **Overall** |
| **1999** | 6.9 (6.7-7.1) | 7.5 (7.3-7.6) | 7.4 (7.2-7.5) |
| **2000** | 7.1 (6.9-7.4) | 7.7 (7.5-7.8) | 7.6 (7.4-7.7) |
| **2001** | 6.9 (6.7-7.1) | 7.7 (7.5-7.8) | 7.5 (7.4-7.6) |
| **2002** | 7.1 (6.9-7.3) | 7.9 (7.7-8) | 7.6 (7.5-7.8) |
| **2003** | 6.7 (6.5-6.9) | 7.4 (7.3-7.6) | 7.2 (7.1-7.3) |
| **2004** | 6.4 (6.2-6.6) | 7.1 (7-7.3) | 6.9 (6.8-7) |
| **2005** | 6.7 (6.5-6.9) | 7.4 (7.2-7.5) | 7.2 (7-7.3) |
| **2006** | 6.4 (6.3-6.6) | 7.3 (7.2-7.5) | 7.1 (7-7.2) |
| **2007** | 6.5 (6.3-6.7) | 7.4 (7.2-7.5) | 7.1 (7-7.2) |
| **2008** | 6.4 (6.2-6.6) | 7.4 (7.2-7.5) | 7.1 (6.9-7.2) |
| **2009** | 6.3 (6.1-6.5) | 7 (6.9-7.1) | 6.8 (6.7-6.9) |
| **2010** | 6.6 (6.5-6.8) | 7.3 (7.2-7.5) | 7.1 (7-7.2) |
| **2011** | 6.7 (6.5-6.9) | 7.5 (7.3-7.6) | 7.2 (7.1-7.4) |
| **2012** | 6.6 (6.5-6.8) | 7.5 (7.4-7.7) | 7.3 (7.2-7.4) |
| **2013** | 6.8 (6.6-6.9) | 7.4 (7.2-7.5) | 7.2 (7.1-7.3) |
| **2014** | 6.8 (6.7-7) | 7.3 (7.1-7.4) | 7.2 (7.1-7.3) |
| **2015** | 6.7 (6.6-6.9) | 7.3 (7.1-7.4) | 7.1 (7-7.2) |
| **2016** | 6.7 (6.6-6.9) | 6.7 (6.6-6.9) | 6.8 (6.7-6.9) |
| **2017** | 6.2 (6-6.3) | 6.2 (6-6.3) | 6.2 (6.1-6.3) |
| **2018** | 5.8 (5.7-6) | 5.6 (5.5-5.8) | 5.8 (5.7-5.9) |
| **2019** | 6.2 (6-6.3) | 5.8 (5.7-5.9) | 6 (5.9-6.1) |
| **2020** | 6.9 (6.7-7) | 6 (5.9-6.2) | 6.4 (6.4-6.5) |
| **Total** | 6.6 (6.6-6.6) | 7.1 (7-7.1) | 7 (6.9-7) |

**Supplemental Table 5: Race‐Stratified Stroke-related Age-Adjusted Mortality Rates per 100,000 in Older Adults with Atrial Fibrillation in the United States, 1999 to 2020**

| **Age-Adjusted Rate (95% CI)** | | | | | |
| --- | --- | --- | --- | --- | --- |
| **Year** | **NH White** | **NH Black or African American** | **NH American Indian or Alaska Native** | **Hispanic or Latino** | **NH Asian or Pacific Islander** |
| **1999** | 7.7 (7.6-7.8) | 5.4 (5.0-5.8) | 4.5 (2.9-6.7) | 3.9 (3.4-4.4) | 5.4 (4.6-6.2) |
| **2000** | 7.9 (7.8-8.1) | 5.5 (5.1-5.9) | 4.1 (2.6-6.1) | 4.3 (3.8-4.8) | 4.8 (4.1-5.6) |
| **2001** | 7.8 (7.7-8.0) | 5.1 (4.8-5.5) | 3.3 (2.1-5.1) | 4.4 (4.0-4.9) | 5.4 (4.6-6.1) |
| **2002** | 8.0 (7.9-8.2) | 5.3 (4.9-5.7) | 4.7 (3.1-6.8) | 4.6 (4.1-5.1) | 5.4 (4.7-6.2) |
| **2003** | 7.5 (7.4-7.7) | 5.5 (5.2-5.9) | 4.6 (3.1-6.6) | 4.0 (3.6-4.4) | 4.9 (4.3-5.6) |
| **2004** | 7.3 (7.2-7.4) | 5.1 (4.7-5.5) | 5.0 (3.4-7.0) | 4.0 (3.6-4.5) | 4.5 (3.9-5.1) |
| **2005** | 7.5 (7.4-7.7) | 5.3 (5.0-5.7) | 3.9 (2.5-5.6) | 4.2 (3.8-4.6) | 5.1 (4.4-5.7) |
| **2006** | 7.4 (7.3-7.6) | 5.0 (4.6-5.3) | 5.9 (4.2-8.0) | 4.1 (3.7-4.5) | 5.5 (4.8-6.1) |
| **2007** | 7.5 (7.3-7.6) | 5.5 (5.1-5.9) | 4.5 (3.1-6.4) | 4.0 (3.6-4.4) | 5.1 (4.5-5.7) |
| **2008** | 7.4 (7.3-7.6) | 5.5 (5.1-5.8) | 4.8 (3.3-6.6) | 4.1 (3.8-4.5) | 4.9 (4.3-5.5) |
| **2009** | 7.2 (7.0-7.3) | 4.9 (4.6-5.3) | 4.0 (2.7-5.7) | 4.3 (3.9-4.6) | 4.6 (4.0-5.1) |
| **2010** | 7.5 (7.3-7.6) | 5.4 (5.0-5.7) | 5.3 (3.8-7.3) | 4.5 (4.2-4.9) | 5.5 (4.9-6.1) |
| **2011** | 7.7 (7.6-7.8) | 5.1 (4.7-5.4) | 5.3 (3.9-7.2) | 4.4 (4.0-4.7) | 4.8 (4.3-5.3) |
| **2012** | 7.7 (7.6-7.8) | 5.7 (5.4-6.1) | 4.7 (3.4-6.3) | 4.5 (4.1-4.8) | 4.5 (4.0-5.0) |
| **2013** | 7.7 (7.6-7.8) | 5.6 (5.2-5.9) | 5.3 (4.0-7.0) | 4.3 (3.9-4.6) | 4.7 (4.2-5.2) |
| **2014** | 7.7 (7.5-7.8) | 5.3 (5.0-5.6) | 5.7 (4.3-7.4) | 4.7 (4.3-5.0) | 4.4 (4.0-4.9) |
| **2015** | 7.7 (7.5-7.8) | 5.5 (5.1-5.8) | 4.3 (3.2-5.7) | 4.4 (4.1-4.8) | 4.3 (3.9-4.7) |
| **2016** | 7.3 (7.2-7.4) | 5.3 (5.0-5.7) | 5.0 (3.8-6.5) | 4.2 (3.9-4.5) | 4.5 (4.0-4.9) |
| **2017** | 6.7 (6.6-6.8) | 5.1 (4.8-5.4) | 4.3 (3.2-5.6) | 3.7 (3.4-4.0) | 3.6 (3.3-4.0) |
| **2018** | 6.2 (6.1-6.3) | 5.0 (4.7-5.3) | 4.7 (3.6-6.1) | 3.4 (3.1-3.6) | 3.3 (3.0-3.6) |
| **2019** | 6.5 (6.3-6.6) | 5.3 (5.0-5.6) | 3.2 (2.3-4.3) | 3.5 (3.3-3.8) | 4.0 (3.6-4.3) |
| **2020** | 6.9 (6.8-7.0) | 6.2 (5.9-6.5) | 4.1 (3.1-5.2) | 4.0 (3.8-4.3) | 3.9 (3.5-4.2) |
| **Total** | 7.4 (7.4-7.4) | 5.4 (5.3-5.4) | 4.6 (4.3-4.9) | 4.1 (4.1-4.2) | 4.5 (4.4-4.6) |
| NH = non-Hispanic. | | | | | |

**Supplemental Table 6: Stroke-related Age-Adjusted Mortality Rates per 100,000, Stratified by States in Older Adults with Atrial Fibrillation, in the United States, 1999 to 2020**

| **State** | **Age-Adjusted Rate (95% CI)** |
| --- | --- |
| Alabama | 7 (6.8-7.2) |
| Alaska | 9.8 (8.9-10.6) |
| Arizona | 4.9 (4.7-5) |
| Arkansas | 5.8 (5.6-6) |
| California | 8.2 (8.1-8.2) |
| Colorado | 6.6 (6.4-6.8) |
| Connecticut | 7.6 (7.4-7.8) |
| Delaware | 6.6 (6.2-7.1) |
| District of Columbia | 6.4 (5.8-6.9) |
| Florida | 4.6 (4.5-4.6) |
| Georgia | 5.3 (5.2-5.4) |
| Hawaii | 6.4 (6.1-6.7) |
| Idaho | 8.4 (8-8.7) |
| Illinois | 5.8 (5.7-5.9) |
| Indiana | 6.6 (6.4-6.8) |
| Iowa | 7.3 (7.1-7.5) |
| Kansas | 5.6 (5.4-5.8) |
| Kentucky | 7.5 (7.3-7.7) |
| Louisiana | 4.6 (4.4-4.8) |
| Maine | 8.4 (8.1-8.8) |
| Maryland | 8.3 (8.1-8.5) |
| Massachusetts | 6.4 (6.3-6.6) |
| Michigan | 5.9 (5.8-6) |
| Minnesota | 8.7 (8.6-8.9) |
| Mississippi | 6.7 (6.5-7) |
| Missouri | 6.1 (6-6.3) |
| Montana | 8 (7.6-8.5) |
| Nebraska | 7.9 (7.6-8.2) |
| Nevada | 4.3 (4.1-4.6) |
| New Hampshire | 8.2 (7.9-8.6) |
| New Jersey | 6.8 (6.6-6.9) |
| New Mexico | 5 (4.7-5.2) |
| New York | 5.2 (5.2-5.3) |
| North Carolina | 8.5 (8.4-8.7) |
| North Dakota | 8.7 (8.2-9.2) |
| Ohio | 8.4 (8.3-8.6) |
| Oklahoma | 8.6 (8.3-8.8) |
| Oregon | 11.8 (11.5-12) |
| Pennsylvania | 7.3 (7.2-7.4) |
| Rhode Island | 9.2 (8.8-9.6) |
| South Carolina | 8.2 (8-8.4) |
| South Dakota | 8.3 (7.9-8.8) |
| Tennessee | 8.4 (8.3-8.6) |
| Texas | 6.8 (6.7-6.9) |
| Utah | 6.5 (6.2-6.8) |
| Vermont | 11.9 (11.3-12.6) |
| Virginia | 6.7 (6.6-6.9) |
| Washington | 10.5 (10.3-10.7) |
| West Virginia | 9.6 (9.3-9.9) |
| Wisconsin | 7.4 (7.2-7.6) |
| Wyoming | 7.4 (6.8-8) |

**Supplemental Table 7: Stroke-related Age-Adjusted Mortality Rates per 100,000, Stratified by Census Region in Older Adults with Atrial Fibrillation in the United States, 1999 to 2020**

|  | **Census Region: Northeast** | **Census Region: Midwest** | **Census Region: South** | **Census Region: West** |
| --- | --- | --- | --- | --- |
| **Year** | **Age-Adjusted Rate (95% CI)** | **Age-Adjusted Rate (95% CI)** | **Age-Adjusted Rate (95% CI)** | **Age-Adjusted Rate (95% CI)** |
| **1999** | 7.2 (6.9-7.5) | 7.2 (6.9-7.4) | 7 (6.8-7.2) | 8.3 (8-8.7) |
| **2000** | 7.3 (7.1-7.6) | 7.4 (7.2-7.7) | 7.2 (7-7.4) | 8.7 (8.4-9) |
| **2001** | 7.3 (7-7.6) | 7.1 (6.9-7.4) | 6.9 (6.7-7.1) | 9.1 (8.8-9.4) |
| **2002** | 7.1 (6.9-7.4) | 7.7 (7.5-8) | 7.3 (7.1-7.6) | 8.6 (8.3-8.9) |
| **2003** | 6.8 (6.5-7) | 6.9 (6.6-7.1) | 6.9 (6.7-7.1) | 8.6 (8.3-8.9) |
| **2004** | 6.5 (6.2-6.7) | 6.7 (6.5-7) | 6.6 (6.4-6.8) | 8.2 (7.9-8.5) |
| **2005** | 6.4 (6.2-6.7) | 7 (6.8-7.2) | 6.8 (6.6-7) | 8.7 (8.4-9) |
| **2006** | 6.5 (6.2-6.7) | 6.8 (6.6-7) | 6.7 (6.5-6.9) | 8.6 (8.3-8.9) |
| **2007** | 6.6 (6.4-6.9) | 7.1 (6.9-7.4) | 6.8 (6.6-7) | 8.2 (7.9-8.5) |
| **2008** | 6.5 (6.2-6.7) | 7.2 (6.9-7.4) | 6.6 (6.4-6.7) | 8.4 (8.1-8.7) |
| **2009** | 6.2 (6-6.4) | 6.7 (6.5-6.9) | 6.4 (6.2-6.6) | 8.1 (7.8-8.4) |
| **2010** | 6.7 (6.5-7) | 7 (6.8-7.3) | 6.7 (6.6-6.9) | 8.2 (7.9-8.4) |
| **2011** | 6.8 (6.6-7.1) | 7.2 (6.9-7.4) | 6.7 (6.5-6.9) | 8.6 (8.3-8.8) |
| **2012** | 7.1 (6.8-7.3) | 7.3 (7.1-7.5) | 6.8 (6.6-7) | 8.2 (8-8.5) |
| **2013** | 6.9 (6.6-7.1) | 7.2 (7-7.4) | 6.8 (6.6-7) | 8.1 (7.8-8.3) |
| **2014** | 7 (6.7-7.2) | 7.1 (6.9-7.3) | 6.7 (6.5-6.9) | 8.1 (7.9-8.4) |
| **2015** | 6.9 (6.7-7.1) | 7.3 (7.1-7.6) | 6.7 (6.5-6.9) | 7.9 (7.7-8.2) |
| **2016** | 6.7 (6.4-6.9) | 6.9 (6.6-7.1) | 6.3 (6.1-6.5) | 7.7 (7.4-7.9) |
| **2017** | 6 (5.8-6.2) | 6.4 (6.2-6.6) | 5.9 (5.8-6.1) | 6.8 (6.6-7) |
| **2018** | 5.4 (5.2-5.6) | 6 (5.8-6.2) | 5.6 (5.5-5.8) | 6 (5.8-6.2) |
| **2019** | 5.6 (5.4-5.8) | 6.2 (6-6.4) | 5.9 (5.7-6.1) | 6.4 (6.2-6.6) |
| **2020** | 6 (5.8-6.2) | 6.9 (6.7-7.1) | 6.3 (6.1-6.4) | 6.6 (6.4-6.8) |
| **Total** | 6.6 (6.5-6.6) | 7 (6.9-7) | 6.6 (6.5-6.6) | 7.9 (7.8-8) |

**Supplemental Table 8: Stroke-related Age-Adjusted Mortality Rates per 100,000, Stratified by Urban-Rural Classification in Older Adults with Atrial Fibrillation in the United States, 1999 to 2020**

| **Age-Adjusted Rate (95% CI)** | | |
| --- | --- | --- |
| **Year** | **Metropolitan** | **Nonmetropolitan** |
| **1999** | 7.2 (7.0-7.3) | 8.1 (7.8-8.4) |
| **2000** | 7.5 (7.3-7.6) | 8.0 (7.7-8.3) |
| **2001** | 7.3 (7.2-7.5) | 8.0 (7.7-8.3) |
| **2002** | 7.4 (7.3-7.6) | 8.5 (8.2-8.8) |
| **2003** | 7.1 (6.9-7.2) | 7.9 (7.6-8.2) |
| **2004** | 6.7 (6.6-6.9) | 7.8 (7.5-8.1) |
| **2005** | 7.0 (6.8-7.1) | 8.0 (7.7-8.3) |
| **2006** | 6.9 (6.8-7.0) | 7.7 (7.4-8.0) |
| **2007** | 6.9 (6.8-7.1) | 7.8 (7.5-8.1) |
| **2008** | 6.9 (6.8-7.0) | 7.8 (7.5-8.0) |
| **2009** | 6.6 (6.5-6.7) | 7.6 (7.3-7.9) |
| **2010** | 6.9 (6.8-7.1) | 7.9 (7.6-8.2) |
| **2011** | 7.1 (6.9-7.2) | 8.0 (7.8-8.3) |
| **2012** | 7.1 (7.0-7.2) | 8.2 (7.9-8.5) |
| **2013** | 7.0 (6.9-7.1) | 8.0 (7.8-8.3) |
| **2014** | 7.0 (6.8-7.1) | 8.3 (8.0-8.5) |
| **2015** | 6.9 (6.8-7.0) | 8.3 (8.1-8.6) |
| **2016** | 6.6 (6.5-6.7) | 7.9 (7.6-8.2) |
| **2017** | 6.0 (5.9-6.1) | 7.6 (7.3-7.8) |
| **2018** | 5.5 (5.4-5.6) | 7.2 (7.0-7.5) |
| **2019** | 5.7 (5.6-5.8) | 7.7 (7.4-7.9) |
| **2020** | 6.2 (6.1-6.3) | 7.9 (7.6-8.2) |
| **Total** | 6.8 (6.7-6.8) | 7.9 (7.8-8) |
